# Exploratory and locomotor activity, learning and memory functions in somatostatin receptor subtype 4 gene-deficient mice in relation to aging and sex

**DOI:** 10.1007/s11357-019-00059-1

**Published:** 2019-03-22

**Authors:** Nikolett Szentes, Valéria Tékus, Violetta Mohos, Éva Borbély, Zsuzsanna Helyes

**Affiliations:** 1 Department of Pharmacology and Pharmacotherapy, Faculty of Medicine, János Szentágothai Research Centre & Centre for Neuroscience, University of Pécs, Szigeti u. 12, Pécs, H-7624 Hungary; 2 PharmInVivo Ltd., Pécs, Hungary; 3 Department of Pharmacology, Faculty of Pharmacy, University of Pécs, Pécs, Hungary

**Keywords:** Somatostatin, sst_4_ receptor, Behavior, Exploratory, Memory, Locomotion

## Abstract

The inhibitory neuropeptide somatostatin regulates several functions in the nervous system including memory. Its concentrations decrease by age leading to functional alterations, but there are little known about the receptorial mechanism. We discovered that somatostatin receptor 4 (sst_4_) mediates analgesic, anti-depressant, and anti-inflammatory effects without endocrine actions, and it is a unique target for drug development. We investigated the exploratory and locomotor activities and learning and memory functions of male and female sst_4_gene-deficient mice compared with their wild-types (WT) at ages of 3, 12, 17 months in the Y-maze test, open field test (OFT), radial-arm maze (RAM) test and novel object recognition (NOR) test. Young sst_4_ gene-deficient females visited, repeated, and missed significantly less arms than the WTs in the RAM; males showed decreased exploration in the NOR. Young mice moved significantly more, spend longer time in OFT center, and visited more arms in the Y-maze than older ones. Young WT females spend significantly longer time in the OFT center, visited, missed and repeated more arms of the RAM than males. Old males found more rewards than females. Young males explored longer the novel object than young females and older males in the NOR; the recognition index was smaller in females. We conclude that aging and sex are important factors of behavioral parameters that should be focused on in such studies. Sst_4_ is likely to influence locomotion and exploratory behavior only in young mice, but not during normal aging, which is a beneficial feature of a good drug target focusing on the elderly.

## Introduction

Aging strongly influences cognitive functions, memory, and learning. Learning slows down, but memory does not necessarily worsen in the aging population that is continuously increasing in the twenty-first century. Identifying targets for cognitive impairment and memory deficits is in the focus of drug development (Martel et al. 2012). Besides aging, sex is also a crucial factor of cognitive performance (Ruan et al. 2017); therefore, it is important to analyze the differences in preclinical models. It is well established in a variety of species including humans (Vedovelli et al. 2017), rats (Casad 1990; Tenk et al. 2017), and mice (Ashpole et al. 2017; Fang et al. 2017; Reglodi et al. 2018; Spik and Sonntag 1989; Ungvari et al. 2017a) that peptide neurotransmitters, such as somatostatin, pituitary adenylate cyclase-activating polypeptide, corticotropin-releasing factor, insulin-like growth factor 1, growth hormone (GH), neurotrophic factor, connective tissue growth factor, play important regulatory roles in age-related diseases, and both their brain and peripheral concentrations change throughout aging.

Somatostatin is a 14 or 28 amino acid-containing peptide with a disulfide bridge discovered in the hypothalamus (Guillemin 1972) and originally described as a GH or somatotropin inhibiting factor (SRIF). Later it was characterized to be a broad spectrum inhibitory neurotransmitter with a complex effect throughout the central nervous system (CNS) (Epelbaum 1986; Martel et al. 2012; Viollet et al. 2008), as well as the periphery to mediate a variety of auto- , para- or endocrine actions (Leblanc et al. 1975; Pintér et al. 2006). Two subpopulations of somatostatinergic neurons can be distinguished in the CNS, long-protruding somatostatinergic neurons and short-proximal glutamate and gamma-aminobutyric acid (GABA)-ergic interneurons (Epelbaum 1986; Gulyás et al. 2003; Tomioka et al. 2005). Somatostatin inhibits the release of several excitatory and inhibitory neurotransmitters, such as serotonin, acetylcholine, glutamate, and GABA (Baraban and Tallent 2004). It plays a role in sensory perception and pain, motor functions, sleep, cognitive performance (Helyes et al. 2009; Matsuoka et al. 1994), and neurodegenerative disorders (Martel et al. 2012; Tuboly and Vecsei 2013), neuroendocrine and emotional regulation, anxiety and depression (Engin et al. 2008; Lin and Sibille 2015). Our team has provided strong proof-of-concept evidence for systemic anti-inflammatory and analgesic effects of somatostatin released from the activated capsaicin-sensitive peptidergic sensory nerves at the periphery called “sensocrine” function (Szolcsányi et al. 2004; Thán et al. 2000).

Brain somatostatin concentrations and its functions are strongly influenced by aging. Early gene expression studies demonstrated a significant reduction of somatostatin mRNA in the striatum, frontal, and parietal cortex, without significant changes in the hypothalamus of female Wistar rats (Florio et al. 1991). Furthermore, hypothalamic somatostatin immunoreactivity significantly decreases in aged female mice (Kuwahara et al. 2004) and rats (Kim and Choe 2018). In the frontal cortex, a certain somatostatinergic neuronal population is remarkably down-regulated (French et al. 2017). Somatostatin-induced GH-release inhibition was more sensitive in old animals (Kim and Choe 2018). Moreover, genetic deletion of somatostatin leads to reduced hippocampal neprilysin inactivity and increased Aβ_42_-formation also in young animals, which suggests a potential protective role of somatostatin in the development of Alzheimer’s disease and cognitive deficits (Saito et al. 2005). Despite all these data about the expressional and functional alterations of the somatostatinergic systems in the aging brain, very little is known about the regulation, sensitivity, and mechanisms of its receptors (Kim and Choe 2018).

The broad range of actions of somatostatin is mediated by its 5 G_i_ protein-coupled receptors (sst_1–5_) categorized into SRIF1 (sst_2, 3, 5_) and SRIF2 (sst_1, 4_) groups on the basis of synthetic agonist binding potentials (Hoyer et al. 1995). The SRIF2 receptors mediate the endocrine effect of somatostatin, while the SRIF1 ones are responsible for the anti-inflammatory, analgesic, anti-anxiety and anti-depressant actions (Prévôt et al. 2017; Scheich et al. 2016, 2017a). We discovered that the sst_4_ receptor is a very promising target to inhibit neurogenic inflammation, neuropathic pain, and depression (Scheich et al. 2016, 2017b), which was supported by others (Schuelert et al. 2015; Shenoy et al. 2018). Therefore, small molecule sst_4_ agonists are potential drug candidates as novel analgesic drugs with simultaneous anti-depressant activity (Botz et al. 2017; Scheich et al. 2016, 2017b). It is very important to elucidate the complex CNS functions of the sst_4_ receptor from this drug development point of view as well.

Although the precise expression of sst_4_ is not known due to the lack of reliable antibodies, data suggest that it is present in the hippocampus, striatum related to behavior, cognition, and memory (Gastambide et al. 2010; Nakagawasai et al. 2003; Schreff et al. 2000; Viollet et al. 1997), and a small molecule agonist was described to improve long-term and short-term learning in a mouse model of neurodegeneration (Gastambide et al. 2009; Sandoval et al. 2011).

Therefore, in the present study, we investigated the locomotor activity, anxiety, and memory functions in male and female mice throughout aging, as well as the role of the sst_4_ receptor on these parameters.

## Materials and methods

### Animals

We examined male and female sst_4_ gene-deficient mice (knockout, KO) (Helyes et al. 2009; Scheich et al. 2016, 2017b) and wild-type (WT) counterpart of three different ages (3, 12, 17-month-old) in different behavioral tests. They were bred and kept in the Laboratory Animal House of the Department of Pharmacology and Pharmacotherapy of the University of Pécs, Medical School. All animals were in standard plastic cages at 24–25 °C, under a 12–12 h light-dark cycle and provided by standard rodent chow and water ad libitum.

All experiments were carried out in accordance with the recommendations of the 1998/XXVIII Act of the Hungarian Parliament on Animal Protection (243/1988) and were approved by the Ethics Committee on Animal Research of Pécs University (license no. BA02/2000-76/2017).

### Y-maze test

This test is suitable for rodent memory and route-learning capabilities, where we investigated the exploratory behavior of the mice for new ways (Holcomb et al. 1999; Hullmann et al. 2017). They were placed in the upper arm of the Y-shaped maze, each arm having the same length (35 cm), width (5 cm) and height (6 cm). Mice could freely move within the 5-min-period, the number of visited arms and the alternation index (%) (*n*(arm combination))/(*n*(total number of visited arms-2))×100 were determined.

### Radial arm maze (RAM) test

This test is appropriate for investigating both short-term (working memory) and long-term (reference memory) memory functions (Frick et al. 1999; Gresack and Frick 2003).

It is constructed of eight arms with a well-defined central region where the mice start from. Each arm is 25 cm long, 7.5 cm wide, and 6 cm tall, the central part is 20 cm in diameter. Four sugar pellets (rewards) were placed in four defined arms, their locations did not change during the experiment. The entire study lasted for 4 days, the measurement time was 5 min every day in the arena. Mice were conditioned on the first 3 days, without and with rewards on the first and second/third days, respectively. The measurement used for evaluation was performed on the fourth day. The test lasted until the animal found all the four sugar pellets, but it was maximized for 5 min (Zhang and O’Donnell 2000). The number of visited, repeated (when the animal reentered a previously visited reward-containing arm), and missed arms (when the animal entered an arm that does not contain rewards), as well as the number of the found rewards and the time to find all the rewards were measured (Astur et al. 2004; Crusio and Schwegler 2005).

We determined the spatial working (the number of repeated arms divided by all visits, then multiplied by 100) and reference (the number of missed arms divided by all visits, then multiplied by 100) memory errors.

### Novel object recognition (NOR) test

This test was carried out in a 40 × 40 cm high-walled box (open-field box) divided into 20 sections and lasted for 3 days. On the first day, the animals are habituated for 5 min, and the test served as an open field test (OFT) when the spontaneous locomotor activity and anxiety (time spent by moving, in the middle and at the periphery) were evaluated (Carola et al. 2002; Gaszner et al. 2012; Scheich et al. 2016). On the second day, two identical objects (smaller than the mouse) were placed in the test area, and the mice were allowed to familiarize with these objects for 5 min. On the third day (after 24 h), one object was replaced with a new one of different shape and color, and the animals have observed it for 5 min. We detected how much time the mice spend with the discovery of the familiar and the novel objects; the ratio of which was determined as the recognition index (Antunes and Biala 2012).

All experiments were recorded by the Noldus system and evaluated by the EthoVision XT software.

### Statistical evaluation

Data are shown as means ± SEM, and factorial ANOVA followed by Tukey’s HSD post hoc test was used for statistical evaluation. Significant differences were highlighted in all figures as follows: *p < 0.05, **p < 0.01, ***p < 0.001 (related to age; older groups vs. young ones); #*p* < 0.05, ##*p* < 0.01, ###*p* < 0.001 (relates to sex; females vs. males); +*p* < 0.05, ++*p* < 0.01, +++*p* < 0.001 (related to genotype; KO vs. WT).

## Results

### Behavior of male and female sst_4_ KO mice of different ages in comparison with respective WT to controls in the Y-maze

**Fig. 1:**
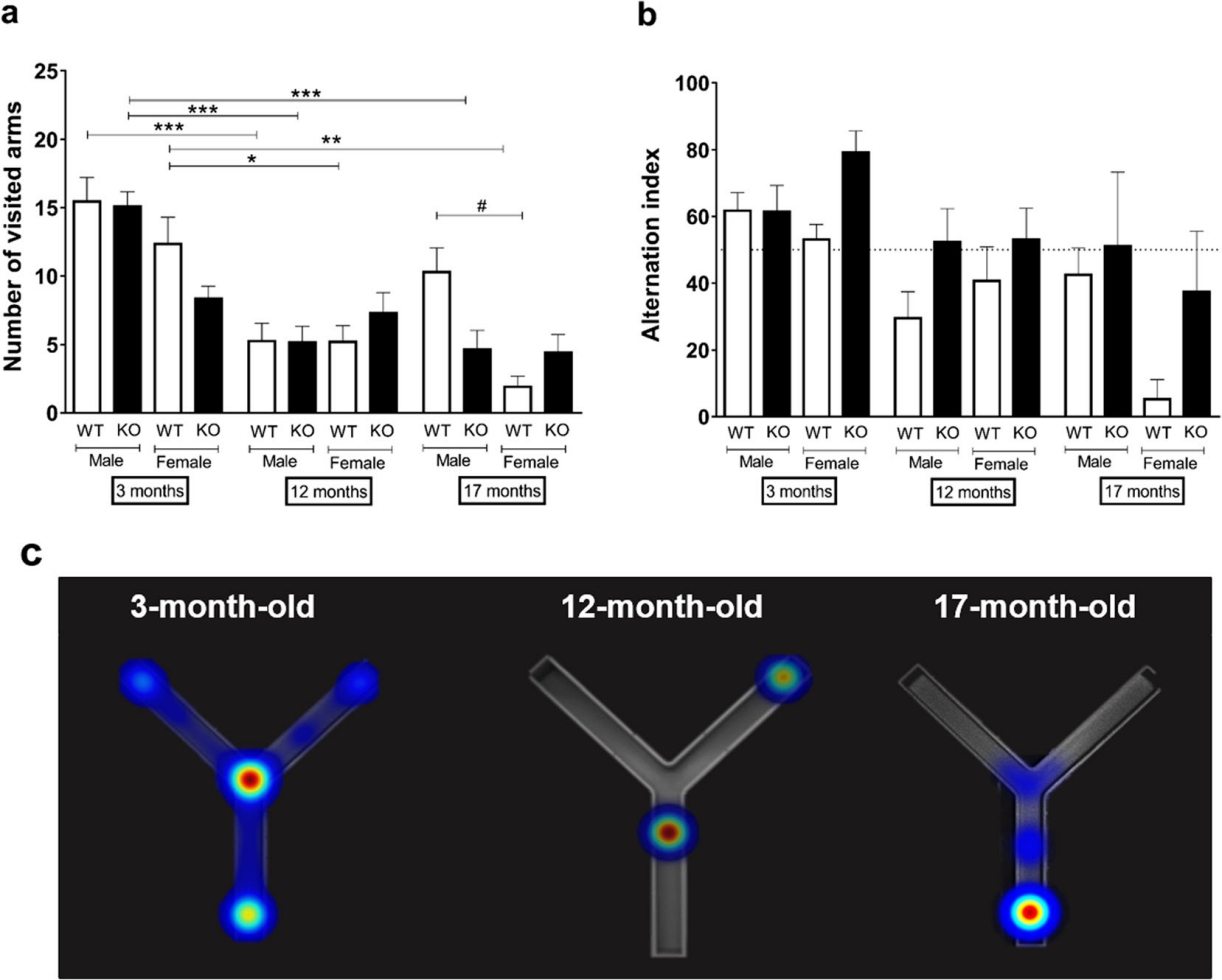
**a** The total number of visited arms and **b** arm combination in the Y-maze test showing spatial working memory of mice by spontaneous alternation of male and female sst_4_ gene-deficient mice and WT counterparts of three different ages (3-, 12-, 17-months-old). **c** Representative heatmap pictures of male KO in the three different age groups. Data are means + SEM, *p < 0.05, **p < 0.01, ***p < 0.001 (vs. age); #*p* < 0.05 (vs. sex); factorial ANOVA followed by Tukey’s HSD post hoc test

Young male and female WT and sst_4_ gene-deficient mice visited significantly more arms than their older counterparts. Furthermore, the 17-month-old female WTs visited significantly less arms compared with males. Deletion of the sst_4_ receptor did not alter the behavior in this test in any age groups either in males or females. There were no significant differences in arm combinations in relation to age, sex, and sst_4_ receptor deletion (Fig. 1).

### Sst_4_ deficiency and aging worsen the RAM performance of female but not male mice

**Fig. 2:**
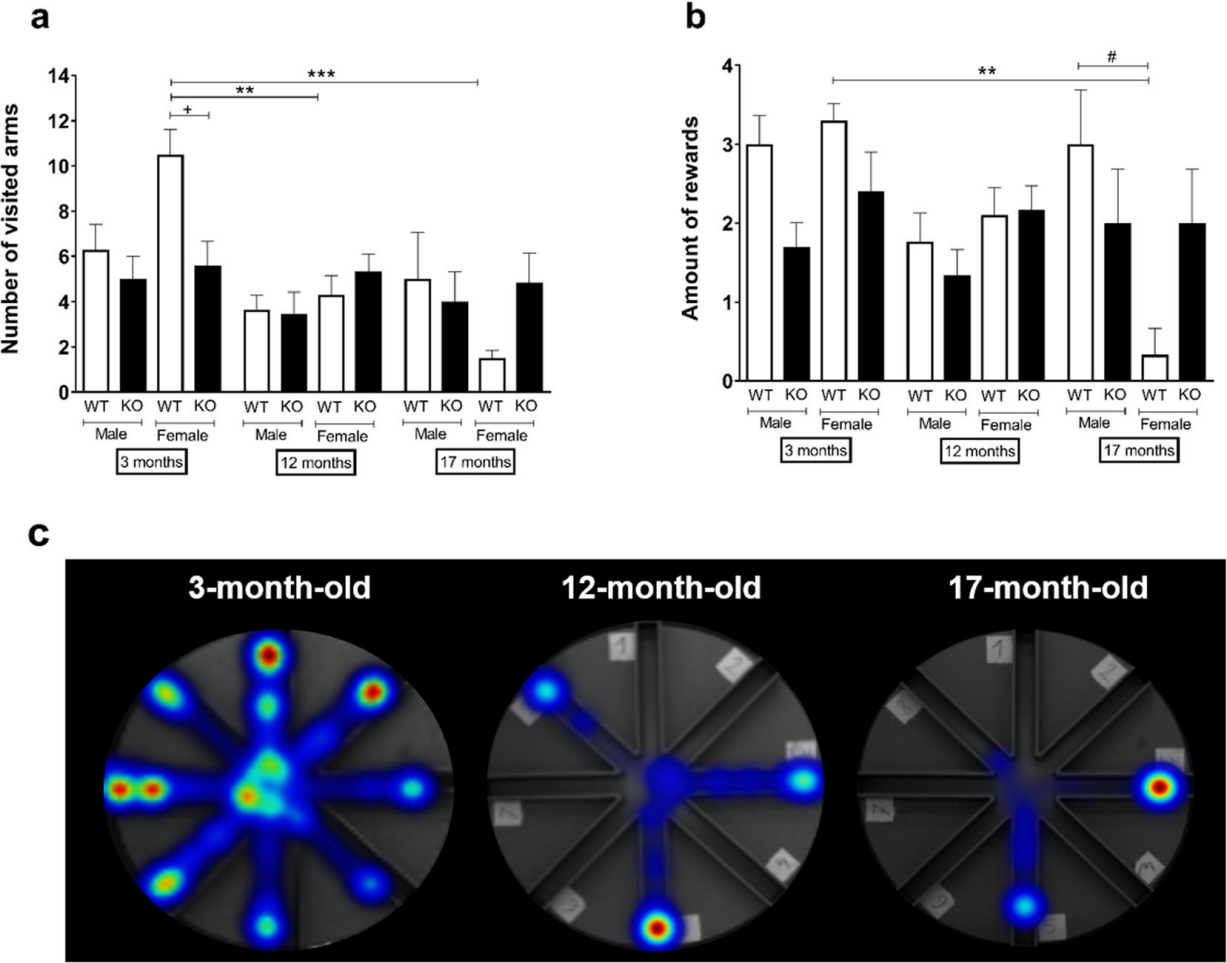
**a** The number of visited, **b** amount of reward found in the RAM. **c** Representative heatmap pictures of female WT in the three different age groups. Data are means + SEM, **p < 0.01, ***p < 0.001 (vs. age); #*p* < 0.05 (vs. sex); +*p* < 0.05 (vs. gene); factorial ANOVA followed by Tukey’s HSD post hoc test

Young sst_4_ gene-deficient female mice visited, repeated, and missed significantly less arms than the WT counterparts. There was a remarkable sex difference in young mice, females repeated and missed significantly more arms than the males, and also visited more arms, although this parameter was not statistically significant. In the female, but not in the male WT group, young mice visited, repeated and missed more and found significantly more rewards than the respective old ones (Fig. 2).

### Working and reference memory functions of male and female sst_4_ KO mice of different ages in comparison with respective WT controls in the RAM

**Fig. 3:**
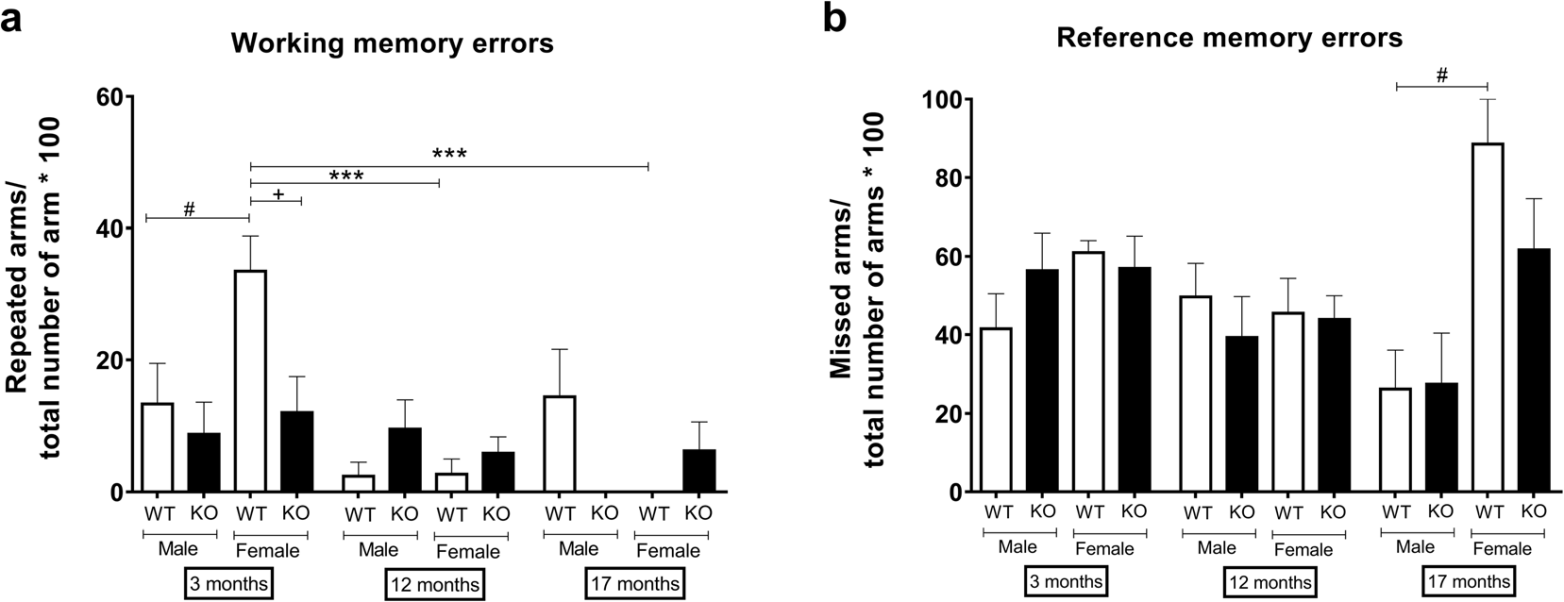
**a** Working and **b** reference memory functions. Data are means + SEM, ***p < 0.001 (vs. age); #*p* < 0.05 (vs. sex); +*p* < 0.05 (vs. gene); factorial ANOVA followed by Tukey’s HSD post hoc test

Surprisingly, both aging and sst_4_ deletion significantly improved the working memory of female but not of male mice. However, the working memory of young and the reference memory of old WT female mice were worse than these functions of age-matched male controls, respectively (Fig. 3).

### Aging decreases spontaneous locomotor activity and increases anxiety level in both sexes independently of the sst_4_ receptor in the OFT

**Fig. 4:**
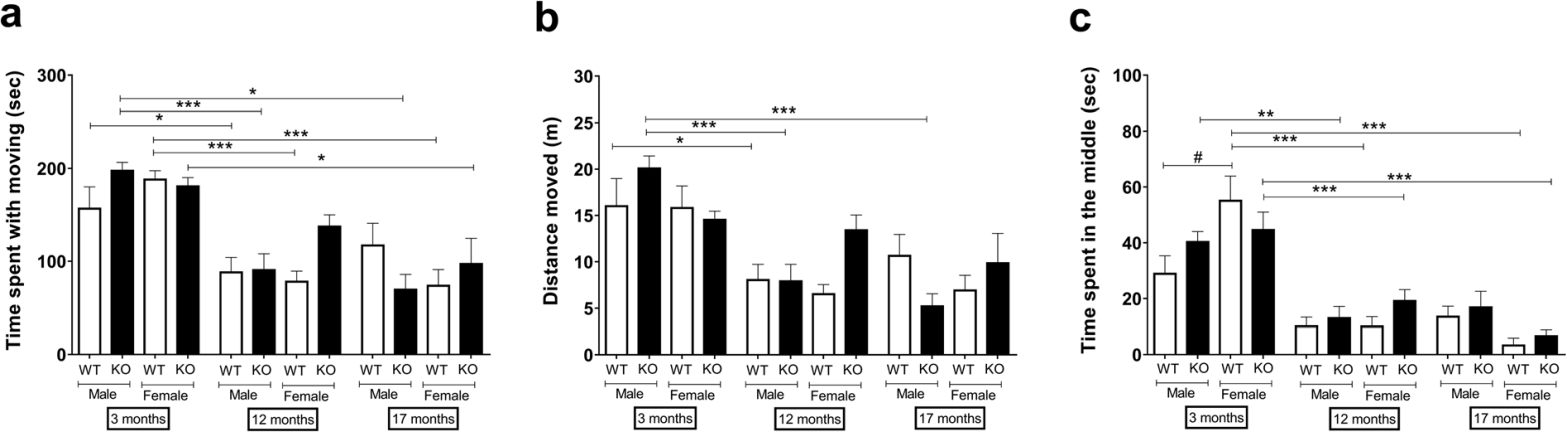
**a** Time spend with moving, **b** distance moved, and **c** time spend in the middle in the open field box. Data are means + SEM, *p < 0.05, **p < 0.01, ***p < 0.001 (vs. age); #*p* < 0.05 (vs. sex); factorial ANOVA followed by Tukey’s HSD post hoc test

The 12- and 17-month-old mice of both sexes and genotypes moved significantly less during the 5-min measurement, spend less time in the middle and more at the periphery of the OF box than their young controls. It is also important to note that in the young WT group, females spend significantly more time in the middle and less at the periphery than the age-matched males (Fig. 4).

### Decreased exploratory behavior in sst_4_-deficient young male, female and aged mice, but unaltered novelty detection and recognition memory functions in the NOR test

Young WT male mice spend significantly more time with both the familiar and novel objects then the sst_4_ gene-deficient ones, as well as the female counterparts and old ones both in cases of the first test (day 2) and the repeated test (day 3) (Fig. 5a–d).

**Figure:**
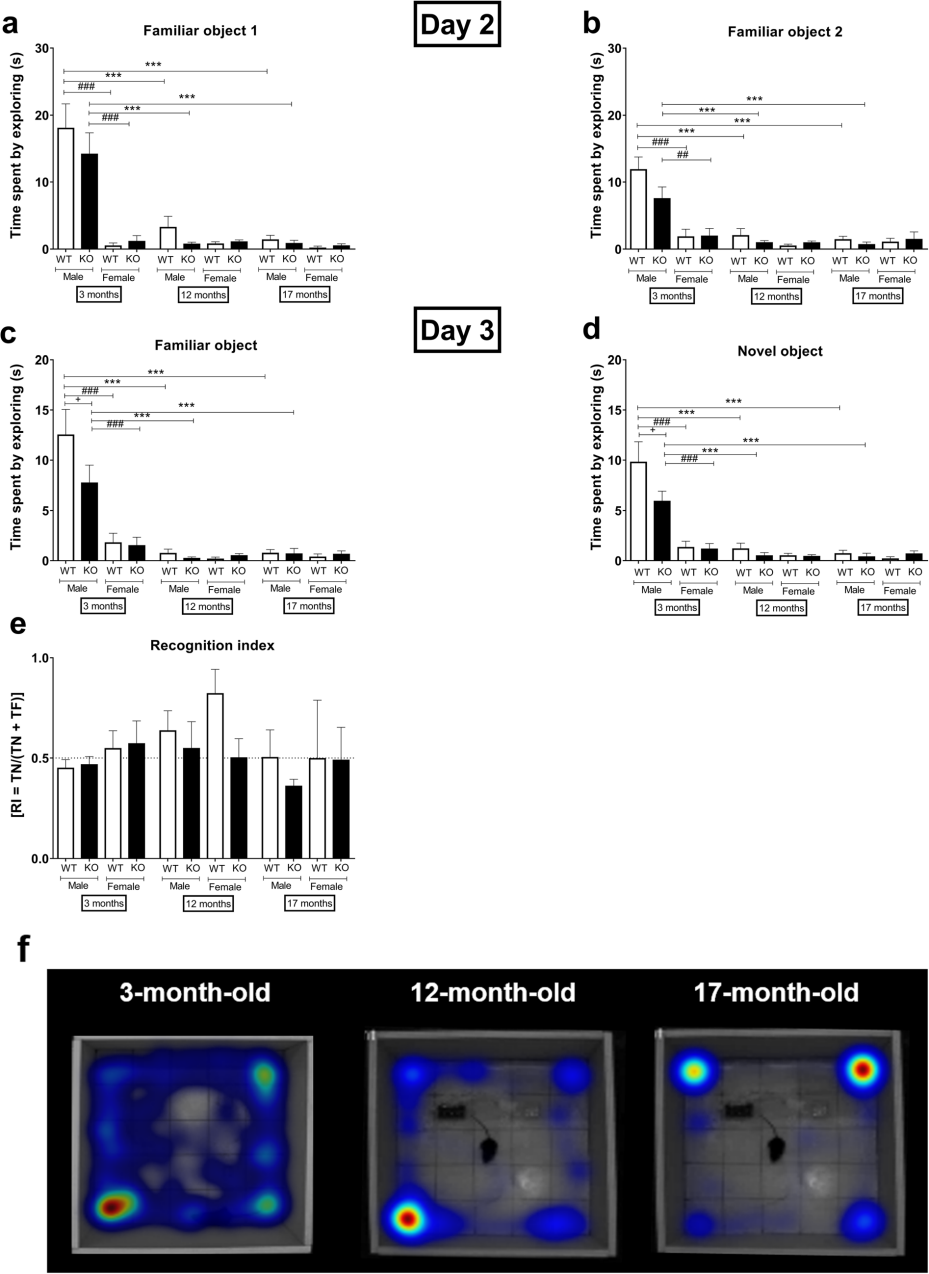

The older, 12-, and 17-month-old mice of both sexes and genotypes were much less interested in exploring both objects. The recognition index determined by the ratio of the novel and the familiar object investigations showing the memory function did not show any difference in any groups (Fig. 5e).

## Discussion

We show here that aging and sex are very important factors of behavioral parameters that have to be focused on in such studies. Furthermore, the somatostatin sst_4_ receptor is likely to be involved in locomotion and exploratory behaviors only in young mice and does not influence behavior during normal aging.

Somatostatin is expressed in brain regions related to pain and mood regulation like the dorsolateral prefrontal, cingulate cortex, and amygdala (Guilloux et al. 2012; Sibille et al. 2011; Tripp et al. 2011). Its important inhibitory functions in several physiological and pathophysiological processes, such as sensory, locomotion and motor coordination (Zeyda et al. 2001), stress-related and learning activities (Viollet et al. 2008), mood regulation (Engin et al. 2008; Lin and Sibille 2015), cognitive performance, and neurodegeneration (Saito et al. 2005) has also been evidenced in animal models. Furthermore, lower somatostatin levels were measured in the cerebrospinal fluid and the brain areas of patients with major and bipolar depressive disorders, schizophrenia, Alzheimer’s, and Parkinson’s diseases (Lin and Sibille 2013).

Somatostatin levels strongly decrease in the aging brain to that mRNA and protein levels (Florio et al. 1991; Kuwahara et al. 2004), and its functions are also altered (French et al. 2017; Kim and Choe 2018).

The expression of the sst_4_ receptor in the brain is similar to that of somatostatin (Martel et al. 2012), but there are no data about its functions and changes in aging. We earlier found enhanced inflammatory and neuropathic hyperalgesia in sst_4_-deleted mice (Helyes et al. 2009). Increased depression-like behaviors and anxiety, as well as altered neuronal activation in the central and basolateral amygdaloid nuclei, were detected in sst_4_ gene-deficient mice upon both acute and chronic stress (Scheich et al. 2017b).

The present results clearly demonstrate that the lack of sst_4_ only influences some behaviors of young mice: females visited, repeated, and missed significantly less arms than the WTs in the RAM, while males showed decreased exploration in the NOR. Since the outcomes of these behavioral tests are greatly modified by the anxiety level of the animals, and the sst_4_-deficient mice have more anxious and depressive-like phenotye (Scheich et al. 2016), our findings might not only be explained by the direct inhibition of learning and locomotion by somatostatin via this receptor, but higher stress level could also be an influencing factor.

Since substantial influence of aging and sex on the behavioral parameters in rodent experiments is well-established (Frick et al. 1999; Sutcliffe et al. 2007), we investigated the impact of these factors in our test systems in order to get a complex picture. The OFT (Carola et al. 2002) and the spontaneously alternating Y-maze test are suitable for determining spontaneous locomotor activity, exploratory behavior, and anxiety level (Borbély et al. 2013). Furthermore, the arm combination in the latter test also refers to working memory functions, since the animal has to remember the two arms that visited for the previous time to get the opportunity to choose and create a new alternative (Hughes 2004). Locomotor activity, anxiety, exploration time in both sexes, but working memory of females declined with age. Young males showed worse locomotor activity, higher anxiety, and better recognition memory, but old ones better working memory than females. Young mice moved significantly more, spend longer time in OFT center, and visited more arms in the Y-maze than older ones of both sexes. As for sex differences, we found that young females were more active than males, but the oldest females were less active and anxious than the respective males.

The influence of aging and sex on behavioral and memory parameters was investigated by others earlier with C57BL/6NIA mice. They found deteriorated memory with aging in both sexes up to 25 months in the Morris water maze, elevated plus maze, OFT that are in agreement with our findings. The 17-month-old mice showed less exploratory behavior, females had higher anxiety level and better spatial reference memory than males (Frick et al. 1999). Cognitive tests were performed in order to assess spontaneous movement, daily activity, distance moved, velocity, and acceleration in a 90-h period of time in young and old (C57B1/6J male 6, 21 and 27 months old) mice. Learning abilities and locomotor activity decreased age dependently and similarly to rats and humans. However, memory decline was not observed in all elderly mice (Logan et al. 2018). Furthermore, in a recent study, cognitive decline was investigated in C57BL/6J mice in the RAM test after brain irradiation which showed that this test is suitable method for assessing memory function in rodents (Ungvari et al. 2017b).

In both the RAM and NOR tests, the working and recognition memory of young animals were better, respectively. The NOR is widely used to examine memory processes (Bevins and Besheer 2006). Young WT females visited, missed, and repeated more arms of the RAM, but old males found more rewards. In the NOR, young males spend longer time by exploring the novel object than both young females and older males, the recognition index was smaller in females. Similarly to our mouse results here, male rats were shown to perform better in the recognition test (Sutcliffe et al. 2007).

There is a strong proof-of-concept that sst_4_ is a valuable target for the development of analgesic and anti-depressant, as well as anti-inflammatory drugs providing a unique tool for the treatment of these common comorbidities particularly in the elderly. Therefore, small molecule sst_4_ agonists with a completely new mechanism of action are under development for chronic neuropathic pain, concomitant mood disorders, and neurogenic inflammation that are still important unmet medical needs (Botz et al. 2017; Pintér et al. 2006; Scheich et al. 2016).

Synthetic sst_4_ agonists inhibit pain, inflammation (Sándor et al. 2006; Schuelert et al. 2015), depression-like behavior (Scheich et al. 2016), and as neurodegeneration and cognitive dysfunction via increasing neprilysin activity leading to decreased cortical Aβ_1–42_ formation in rodent models (Sandoval et al. 2011, 2012). We can conclude from the present results, that sst_4_ does not influence these functions during normal aging without more severe neuronal damage. Therefore, sst_4_ agonists, as novel drug candidates, are not likely to have a major influence on locomotion and learning ability.
